# Sex differences in brain functional connectivity of hippocampus in mild cognitive impairment

**DOI:** 10.3389/fnagi.2022.959394

**Published:** 2022-08-10

**Authors:** Jordan Williamson, Andriy Yabluchanskiy, Peter Mukli, Dee H. Wu, William Sonntag, Carrie Ciro, Yuan Yang

**Affiliations:** ^1^Neural Control and Rehabilitation Laboratory, Stephenson School of Biomedical Engineering, University of Oklahoma, Norman, OK, United States; ^2^Vascular Cognitive Impairment and Neurodegeneration Program, Oklahoma Center for Geroscience and Healthy Brain Aging, Department of Biochemistry and Molecular Biology, University of Oklahoma Health Sciences Center, Oklahoma City, OK, United States; ^3^Department of Radiological Science and Medical Physics, University of Oklahoma Health Science Center, Oklahoma City, OK, United States; ^4^Data Institute for Societal Challenges, University of Oklahoma, Norman, OK, United States; ^5^School of Computer Science, Gallogly College of Engineering, University of Oklahoma, Norman, OK, United States; ^6^School of Electrical and Computer Engineering, Gallogly College of Engineering, University of Oklahoma, Norman, OK, United States; ^7^Department of Rehabilitation Sciences, University of Oklahoma Health Science Center, Oklahoma City, OK, United States; ^8^School of Electrical and Computer Engineering, University of Oklahoma, Tulsa, OK, United States; ^9^Department of Physical Therapy and Human Movement Sciences, Northwestern University, Chicago, IL, United States

**Keywords:** mild cognitive impairment, sex difference, hippocampus, functional connectivity, Alzheimer’s disease

## Abstract

Mild cognitive impairment (MCI) is the prodromal stage of Alzheimer’s Disease (AD). Prior research shows that females are more impacted by MCI than males. On average females have a greater incidence rate of any dementia and current evidence suggests that they suffer greater cognitive deterioration than males in the same disease stage. Recent research has linked these sex differences to neuroimaging markers of brain pathology, such as hippocampal volumes. Specifically, the rate of hippocampal atrophy affects the progression of AD in females more than males. This study was designed to extend our understanding of the sex-related differences in the brain of participants with MCI. Specifically, we investigated the difference in the hippocampal connectivity to different areas of the brain. The Resting State fMRI and T2 MRI of cognitively normal individuals (*n* = 40, female = 20) and individuals with MCI (*n* = 40, female = 20) from the Alzheimer’s Disease Neuroimaging Initiative (ADNI) were analyzed using the Functional Connectivity Toolbox (CONN). Our results demonstrate that connectivity of hippocampus to the precuneus cortex and brain stem was significantly stronger in males than in females. These results improve our current understanding of the role of hippocampus-precuneus cortex and hippocampus-brainstem connectivity in sex differences in MCI. Understanding the contribution of impaired functional connectivity sex differences may aid in the development of sex specific precision medicine to manipulate hippocampal-precuneus cortex and hippocampal-brainstem connectivity to decrease the progression of MCI to AD.

## Introduction

According to the CDC, there are 6.2 million people in the United States living with Alzheimer’s Disease (AD) in 2021 (Centers for Disease Control and Prevention, 2021). This disease disproportionately affects females as they constitute more than two-thirds of the AD population (Snyder et al., 2016). The higher prevalence of AD in females has been attributed to females having greater longevity compared to males (Guerreiro and Bras, 2015). Since age is the greatest risk factor for the development of AD, it would be reasonable to state that more females would live long enough to develop AD. However, increasing evidence suggests there are other factors contribute to the sex-specific risk of AD such as genetics, hormonal differences, rate of depression, education level, and sleep disturbances (Andrew and Tierney, 2018; Mielke, 2019; Pearce et al., 2022).

The most important predictor is mild cognitive impairment (MCI) that always precedes AD, usually years before meeting the diagnostic criteria of clinical dementia (Petersen, 2004). MCI is defined as cognitive decline greater than expected for a given age but does not notably interfere with daily activities (Salmon, 2011). Current clinical evidence demonstrates about a 20% annual conversion rate of MCI to AD and that more than half of the individuals with MCI progress to dementia within 5 years (Gauthier et al., 2006; Davatzikos et al., 2011; López et al., 2020; McGrattan et al., 2022). In addition to prevalence differences, females experience greater cognitive deterioration than males in the same disease stage (Alzheimer’s Association, 2016) that are also present in individuals with MCI (Sohn et al., 2018). Compared to males with AD, females perform worse on a variety of neuropsychological tasks and have greater total brain atrophy and temporal lobe degeneration (Henderson and Buckwalter, 1994; Chapman et al., 2011; Gumus et al., 2021). Magnetic resonance imaging (MRI) data collected through the Alzheimer’s Disease Neuroimaging Initiative (ADNI) study attested to the faster atrophic rate (Hua et al., 2010). The hippocampus is also known to be affected at the earliest stages of MCI, even before a diagnosis can be made (Braak and Braak, 1995), and hippocampal atrophy has been found to affect the progression of AD only in females (Burke et al., 2019). Recent research revealed additional brain imaging markers that may also contribute to the sex differences in AD and are specifically present in individuals with MCI and that reduced hippocampal volume and any microhemorrhage, regardless of location, are the best MRI features to predict the transition from pre-MCI to MCI (Ferretti et al., 2018; Jiang et al., 2022). Cavedo et al. (2018) found that males with MCI had a higher anterior cingulate cortex amyloid load and glucose hypometabolism in the precuneus, posterior cingulate, and inferior parietal cortex. Similar findings have been reported among cognitively normal adults (Rahman et al., 2020) suggesting that males have a higher brain resilience. However, the role of sex-related differences in hippocampal connectivity during MCI has not been elucidated yet.

This study was designed to extend the understanding of the mechanism underlying the sex differences in pathophysiological biomarkers in individuals with MCI. Our hypothesis was that hippocampal functional connectivity (FC) to the precuneus cortex and the brain stem shows sex-and MCI-specific differences. The FC of the hippocampus will be analyzed and compared between females and males with MCI, as well as cognitively normal females and males as controls.

## Materials and methods

### Data source

The data for this study were extracted from the ADNI^1^, which is a publicly accessible dataset available at adni.loni.usc.edu. Launched in 2003, ADNI is a longitudinal, multi-site, cohort study, led by Principal Investigator Michael W. Weiner, MD. The original study, ADNI-1, has been extended three times and the database contains subject data from ADNI-1, ADNI-GO, ADNI-2, and ADNI-3. The overall goal of the studies was to evaluate whether serial magnetic resonance imaging (MRI), positron emission tomography (PET), other biological markers, and clinical and neuropsychological assessment can be combined to measure the progression of mild cognitive impairment (MCI) and early Alzheimer’s disease (AD). For up-to-date information, see www.adni-info.org.

### Screening process

The data were screened for subjects with MCI. To eliminate multiple images from the same subject, the data included early MCI (EMCI), late MCI (LMCI), or MCI from the 1-year subject visit of ADNI-1, ADNI-GO, ADNI-2, and ADNI-3. Subjects’ selection was also limited to those with data collected from resting-state functional magnetic resonance imaging (rs-fMRI) and 3.0-Tesla T2 magnetic resonance imaging. A similar search methodology was applied for cognitively normal (CN) subjects. The screening resulted in a total of 40 MCI females, 42 MCI males, 25 CN females, and 20 CN males. To balance the number of subjects in each group, 20 of each group were randomly selected for the study. Demographics of MCI subjects are provided in Table 1. This includes age, Apolipoprotein E (ApoE) genotype, the Mini Mental State Examination (MMSE), the Geriatric Depression (GD) Scale, the Global Clinical Dementia Rating (CDR), and the Functional Activities Questionnaire (FAQ), and the Neuropsychiatric Inventory Questionnaire (NPI-Q). IBM SPSS (IBM Corp. Armonk, NY, United States) was used to run independent *t*-tests to ensure there was not a statistically significant sex difference in age, MMSE, GD Scale, CDR, FAQ and NPI-Q (*P* > 0.05). If normal distribution could not be assumed based on the Shapiro–Wilk test, a non-parametric Mann–Whitney test was performed. These values are provided in Table 1.

**TABLE 1:** Mild cognitive impairment subject demographics.

ID	Sex	Age	ApoE genotype	MMSE	GD Scale	CDR	FAQ	NPI-Q
S001	F	74	ε3 ε3	26	6	0.5	0	3
S002	F	65	ε4 ε4	25	1	0.5	1	1
S003	F	71	ε4 ε4	29	0	0.5	0	0
S004	F	80	ε3 ε3	25	1	0.5	0	1
S005	F	70	ε3 ε3	30	5	0.5	0	-
S006	F	65	ε4 ε4	27	7	1.0	30	10
S007	F	79	ε3 ε3	29	0	0.5	4	2
S008	F	58	ε3 ε4	30	1	0.5	0	3
S009	F	76	ε3 ε4	26	7	0.5	4	8
S010	F	61	ε3 ε3	29	3	0.5	5	0
S011	F	72	ε3 ε4	28	2	1.0	19	16
S012	F	72	ε3 ε3	28	5	0.5	0	0
S013	F	84	ε3 ε3	28	6	0.5	8	0
S014	F	69	ε3 ε3	26	1	0.5	0	0
S015	F	72	ε3 ε3	30	2	0.5	0	3
S016	F	72	ε3 ε4	28	0	0.5	6	4
S017	F	81	ε3 ε4	25	2	0.5	7	3
S018	F	77	ε3 ε3	29	1	0.5	0	2
S019	F	67	ε3 ε3	29	2	0.5	0	0
S020	F	63	ε3 ε3	29	1	0.5	1	1
S021	M	68	ε3 ε4	29	0	0.5	2	3
S022	M	72	ε3 ε4	29	0	0.5	12	4
S023	M	62	ε4 ε4	29	0	0.5	0	0
S024	M	58	ε3 ε3	25	0	0.5	1	2
S025	M	74	ε3 ε4	28	2	0.5	3	2
S026	M	63	ε2 ε3	30	1	0.5	1	2
S027	M	90	ε3 ε3	26	2	0.5	4	11
S028	M	86	ε3 ε3	25	1	0.5	6	3
S029	M	87	ε3 ε4	29	1.	1.0	10	12
S030	M	70	ε2 ε4	28	2	0.5	2	8
S031	M	74	ε2 ε3	30	3	0.5	0	2
S032	M	75	ε3 ε4	27	5	1.0	21	7
S033	M	69	ε3 ε3	27	1	0.5	0	1
S034	M	74	ε3 ε3	29	2	1	0	0
S035	M	77	ε2 ε3	28	6	0.5	7	8.0
S036	M	80	ε3 ε4	21	3	1.0	22	4
S037	M	73	ε3 ε4	30	2	0.5	2	2
S038	M	76	ε3 ε3	30	1	0.5	1	1
S039	M	62	ε4 ε4	27	5	0.5	3	7
S040	M	76	ε3 ε3	23	5	0.5	3	4
**Female μ ± SD**		**71 ± 7.1**	-	**27.7 ± 1.7**	**2.5 ± 2.4**	**0.55 ± 0.16**	**4.4 ± 7.7**	**3.0 ± 4.1**
**Male μ ± SD**		**73 ± 8.5**	-	**27.5 ± 2.5**	**2.1 ± 1.9**	**0.6 ± 0.21**	**5.0 ± 6.5**	**4.1 ± 3.5**
**Between sex *t*-tests**		***P* = 0.44**	-	***P* = 0.95**	***P* = 0.58**	***P* = 0.38**	***P* = 0.22**	***P* = 0.12**

Bold values represented by Mean±STD and *p*-values.

### Analysis of functional connectivity and statistical testing

The subject’s original rs-fMRI and MRI images (NiFTI format) were imported into the NITRC Functional Connectivity Toolbox (CONN) version 20b (Whitfield-Gabrieli and Nieto-Castanon, 2012). CONN utilizes SPM12 (Welcome Department of Cognitive Neurology, United Kingdom) and MATLAB R2020a (MathWorks, Natick, MA, United States) in its processes and by default a combination of the Harvard-Oxford atlas (HOA distributed with FSL^2^) (Smith et al., 2004; Woolrich et al., 2009; Jenkinson et al., 2012) and the Automated Anatomical Labeling (AAL) atlas (Tzourio-Mazoyer et al., 2002).

The images were processed through the default functional and structural preprocessing pipeline as detailed in Nieto-Castanon (2020). This included realignment, slice timing correction, coregistration/normalization, segmentation, outlier detection, and smoothing. Additionally, this step extracted the blood-oxygen-level dependent (BOLD) time series from the regions of interest (ROIs) and at the voxels. Next, the images were denoised to remove confounding effects from the BOLD signal through linear regression and band-pass filtering. A quality assurance check was made after the denoising to ensure normalization and that there were no visible artifacts in the data.

A seed-to-voxel analysis was conducted for each subject. This analysis created a seed-based connectivity (SBC) map between the ROI (left or right hippocampus) to every voxel of the brain. The SBC map is computed as the Fisher-transformed bivariant correlation coefficients between the ROI BOLD time series and each individual voxel BOLD time series (Whitfield-Gabrieli and Nieto-Castanon, 2012). The mathematical relationship to construct the SBC is shown below


$$r\,\left(x\right)=\frac{\int S\,\left(x,t\right)\,R\,\left(t\right)\,dt}{{\left(\int R^{2}\,\left(t\right)\,dt\,\int S^{2}\,\left(x,t\right)\,dt\right)}^{1/2}}$$

$$Z\,\left(x\right)=t\,a\,n\,h^{-1}\,\left(r\,\left(x\right)\right)$$

where R is the average ROI BOLD timeseries, S is the BOLD timeseries at each voxel, r is the spatial map of Pearson correlation coefficients, and Z is the SBC map of the Fisher-transformed correlation coefficients for the ROI. Finally, *F*-tests were conducted between the SBC maps to compare differences between groups. For a cortical area to be considered significant, the toolbox used the Gaussian Random Field theory parametric statistics, with a cluster threshold *p* < 0.05 (FDR-corrected) and voxel threshold *p* < 0.001 (uncorrected) to control the type I error in multiple comparisons (Worsley et al., 1996). Additionally, the area must have been over 800 voxels large or cover more than 80 percent of a given atlas (specific brain area).

## Results

The brain regions identified to be significantly different between the MCI and CN groups are shown in Table 2. The left and right para hippocampal gyrus, hippocampus, and amygdala all had significant between-group differences in both sexes. The regions that had a sex-specific were the Precuneus Cortex and the Brainstem, observed only in males.

**TABLE 2:** Brain regions with a significant difference between mild cognitive impairment and cognitively normal for each sex.

Sex	ROI	Brain area (Atlas)	% Atlas covered	# Of voxels
Female (FMCI v FCN)	Right Hippocampus	Left Posterior Para Hippocampal Gyrus	89%	346
		Right Posterior Para Hippocampal Gyrus	89%	283
		Right Hippocampus	100%	342
		Left Hippocampus	94%	318
		Right Amygdala	100%	342
		Left Amygdala	97%	318
	Left Hippocampus	Left Posterior Para Hippocampal Gyrus	91%	354
		Right Posterior Para Hippocampal Gyrus	90%	288
		Right Hippocampus	98%	684
		Left Hippocampus	100%	761
		Right Amygdala	94%	322
		Left Amygdala	100%	327
Male (MMCI v MCN)	Right	Brain Stem	24%	1001
	Hippocampus	Precuneus Cortex	18%	993
		Left Posterior Para Hippocampal Gyrus	97%	380
		Right Posterior Para Hippocampal Gyrus	97%	308
		Right Hippocampus	98%	685
		Left Hippocampus	100%	760
		Right Amygdala	100%	342
		Left Amygdala	100%	327
	Left	Brain Stem	20%	829
	Hippocampus	Precuneus Cortex	20%	1132
		Left Posterior Para Hippocampal Gyrus	92%	358
		Right Posterior Para Hippocampal Gyrus	94%	299
		Right Hippocampus	98%	685
		Left Hippocampus	100%	760
		Right Amygdala	99%	337
		Left Amygdala	100%	327

**FIGURE 1:**
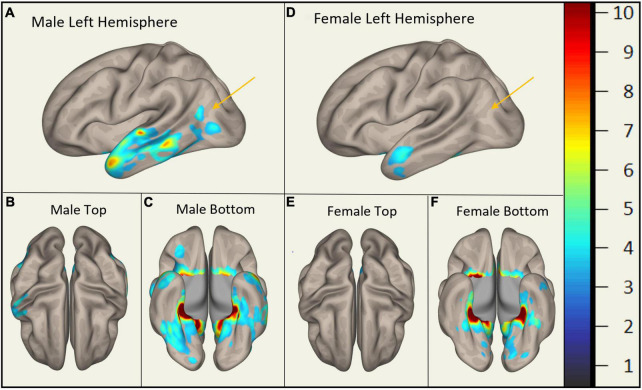
Sex-Specific Pathological Features with Right Hippocampus as ROI. Highlighted display the statistically significant cortical regions between mild cognitive impairment (MCI) and cognitively normal (CN) (*p* < 0.001) normalized to a 1–10 scale. Orange arrows indicate the areas of difference at the precuneus cortex. Panels **(A–C)** display MMCI v MCN. Panels **(D–F)** display FMCI v FCN.

**FIGURE 2:**
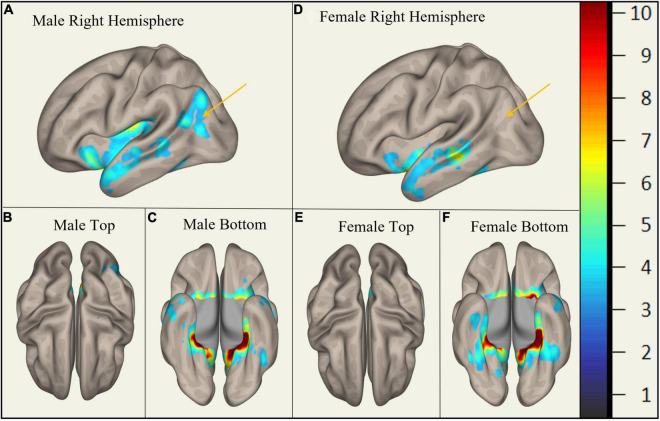
Sex-Specific Pathological Features with Left Hippocampus as ROI. Highlighted areas display the statistically significant cortical regions between mild cognitive impairment (MCI) and cognitively normal (CN) (*p* < 0.001) normalized to a 1–10 scale. Orange arrows indicate the area of difference at the precuneus cortex. Panels **(A–C)** display MMCI v MCN. Panels **(D–F)** display FMCI v FCN.

**FIGURE 3:**
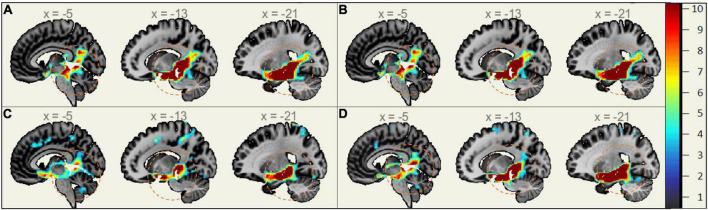
Sex-Specific Pathological Features Sagittal View. Highlighted Areas display the statistically significant regions between cognitively normal (CN) and mild cognitive impairment (MCI) (*p* < 0.001) normalized to a 1–10 scale. Orange circles indicate the area of difference in the brain stem and provide size reference between subplots. **(A)** Right Hippocampus ROI MMCI v MCN. **(B)** Left Hippocampus ROI MMCI v MCN. **(C)** Right Hippocampus ROI FMCI v FCN. **(D)** Left Hippocampus ROI FMCI v FCN.

In MCI, males showed significantly stronger connectivity of the right or left hippocampus to the left or right precuneus cortex, respectively. This difference is shown visually by comparing boxes A and D (see Figures 1–3). There was also a sex specific difference detected in the brain stem. This is visualized in Figure 3.

## Discussion

This study supports that there are sex differences in pathophysiological biomarkers of the brain in MCI. Specifically, it extends our current understanding of the role of the hippocampus in these differences. We demonstrate that hippocampal functional connectivity differs to the precuneus cortex and the brain stem between males and females.

The differences found between the MCI and cognitively normal groups across sexes (posterior para hippocampal gyrus, hippocampus, and amygdala) are consistent with prior studies. The posterior para hippocampal gyrus is the cortical ridge in the medial temporal lobe. It contains the hippocampus (covering it medially) and amygdala (covering it anteromedially) (Goel, 2015). These structures are highly integrated and significant in the process of associative memory (Weniger et al., 2004). It has been shown that functional connectivity between the hippocampus and amygdala to different regions of the brain is disrupted in MCI (Wang et al., 2011; Ortner et al., 2016). This is consistent with our findings.

The role of the precuneus cortex is consistent with other literature highlighting its importance in the development of AD. The precuneus cortex is in the posteromedial portion of the parietal lobe. This area has a central role in a wide range of integrated tasks, including visuo-spatial imagery, episodic memory retrieval, and self-processing operations (Cavanna and Trimble, 2006). The precuneus cortex has been shown to have significantly greater activation in MCI, compared to controls, during visual encoding memory tasks (Rami et al., 2012). Prior studies have shown that functional connectivity between the hippocampus and precuneus cortex differs between individuals with early AD and healthy controls (Kim et al., 2013; Yokoi et al., 2018). However, these studies do not extend to differences between sexes. It has been shown that in individuals with subjective memory complaints, males compared to females had glucose hypometabolism in the precuneus cortex (Cavedo et al., 2018). Our findings extend this knowledge of differences between males and females in the precuneus cortex and show that the effect of MCI on the hippocampal-precuneus cortex functional connectivity may be contributing to the high prevalence of MCI in females.

Previous studies observed that functional connectivity of the locus coeruleus (LC) and the ventral tegmental area (VTA) in the midbrain of the brain stem differ in individuals with AD and MCI. Specifically, the connectivity between the VTA and the para hippocampal gyrus and cerebellar vermis were associated with the occurrence of neuropsychiatric symptoms of AD (Serra et al., 2018). Other studies showed that reduced connectivity between the LC and para hippocampal gyrus in MCI was correlated with memory performance (Jacobs et al., 2015). The difference in functional connectivity seen between males and females in this study extends these known connectivity differences seen between MCI and controls to an additional sex difference. This may be a factor in the observed worse neuropsychological tasks seen in females.

The sex differences observed in MCI have also been attributed to other factors besides functional connectivity. For example, cognitive reserve, referring to education and premorbid intelligence (IQ), is associated with the progression of MCI to AD (Osone et al., 2014). Furthermore, Giacomucci et al. (2022) reported that sex interacts with cognitive reserve and influences the onset and severity of subjective cognitive decline. Additionally, sex differences in the progression of AD from MCI have been correlated with the ApoE ε4 allele, a well-known risk factor for AD. It has been observed that ApoE ε4 is only significantly correlated to the progression of AD in females (Kim et al., 2015).

In summary, these findings are significant as they expand our current understanding of the role of the hippocampus-precuneus cortex and hippocampus-brainstem connectivity in sex differences in MCI. Understanding these sex differences in pathophysiology may aid in the development of sex-specific precision medicine to manipulate hippocampal-precuneus cortex and hippocampal-brainstem connectivity to decrease the progression of MCI to AD. Our findings provide the rationale for sex-specific interventions such as cognitive training (Hardcastle et al., 2022) and neuro-navigation guided, targeted non-invasive brain stimulation (Mackenbach et al., 2020; Yang et al., 2021) or their combination (Vecchio et al., 2022).

*Limitations and Future Work* are related to this study’s number of subjects. While this research provides preliminary findings on sex differences in functional connectivity of the hippocampus in individuals with MCI, the small sample size (*n* = 80) is a limitation. Therefore, future work includes increasing sample size in a larger database, as well as expanding functional connectivity from other regions of interest for MCI, in addition to the hippocampus. Furthermore, studies such as these could be furthered by combining mentioned risk factors such as cognitive reserve or genetic differences to explore if there is any connection.

## Data availability statement

The original contributions presented in this study are included in the article/supplementary material, further inquiries can be directed to the corresponding author/s.

## Author contributions

JW conducted the study and drafted the manuscript. YY contributed to conceptualization, problem solving, and guidance during the conduction of the study. AY, PM, DW, WS, CC, and YY participated in editing the manuscript. All authors contributed to the article and approved the submitted version.

## Footnotes

1
https://adni.loni.usc.edu/

2
http://www.fmrib.ox.ac.uk/fsl/
